# F209. TRANSCRANIAL DIRECT CURRENT STIMULATION (TDCS) IN A NON-CLINICAL POPULATION AS A MODEL FOR TREATMENT OF AUDITORY VERBAL HALLUCINATIONS IN SCHIZOPHRENIA

**DOI:** 10.1093/schbul/sby017.740

**Published:** 2018-04-01

**Authors:** Marquardt Lynn, Isabella Kusztrits, Alex Craven, Kenneth Hugdahl, Marco Hirnstein

**Affiliations:** 1 University of Bergen; 2 University of Bergen, NORMENT Centre of Excellence, University of Oslo, Haukeland University Hospital

## Abstract

**Background:**

We used transcranial direct current stimulation (tDCS) in a non-clinical population to simulate a model of tDCS-treatment for auditory verbal hallucinations (AVH) in schizophrenia. In tDCS, a low current is induced via two electrodes attached to the scalp. The anode and cathode typically up- and downregulate neuronal activity, respectively. It was suggested that AVH arise due to two main neuronal pathways: hyper-activity in the language areas in the temporo-parietal cortex and hypo-activity of the cognitive control areas in the dorsolateral prefrontal cortex. Accordingly, it was further hypothesized that by reducing activity in the temporo-parietal cortex through cathodal tDCS and simultaneously increasing neuronal activity in the dorsolateral prefrontal cortex with anodal tDCS, AVH could be reduced. Patients with schizophrenia, particularly those with AVH show additionally deficits on language and cognitive control tasks, which are known to draw on temporo-parietal and dorsolateral prefrontal cortex regions, respectively. In order to test the model we thus reversed the electrode montage in non-clinical participants and tested whether they would show similar deficits as schizophrenia patients. In addition, the healthy participants underwent magnetic resonance spectroscopy (MRS) to test whether, in accordance with the model, glutamate levels increase under the anode, and decrease under the cathode area.

**Methods:**

Eighteen participants were recruited in a convenience sample (7 male/ 11 female) with a mean age of 26 years. They were tested twice with a mean interval of 8 days. In one session they received real 2mA tDCS for 20 min, while in an MRI scanner (GE 750, 3T). The other session was a sham stimulation control. The order of real/sham stimulation was counterbalanced and stimulation was double-blind. In each session, MRS was measured using a PRESS sequence (TE=35ms, 1500ms) before and after stimulation. MRS data were acquired from two voxels, one in the left dorsolateral prefrontal cortex (22ml) and one in the left temporo parietal cortex (25ml), right underneath anode and cathode electrodes, respectively. MRS data were analyzed using LCModel software; water-scaled estimates for glutamate and glutamine combined (Glx) are reported herein, with N-Acetylaspartate (NAA) and creatine (Cre) inspected to ensure stability of the Glx measure. Glx levels were subjected to a 2x2x2 ANOVA with the within-participant factors Stimulation (real vs sham), Stimulation area (dorsolateral prefrontal cortex versus temporo-parietal cortex), and Time (Pre and Post stimulation).

**Results:**

Two datasets where excluded from analysis due to poor spectral quality. As expected, NAA (F1,16=.809, p=.382) and Cre (F1,16=.005, p=.944) did not show significant changes. There was a trend for Glx to be higher during real as compared to sham stimulation (main effect Stimulation F1,16=3.867, p=.067) and for Glx to be higher after than before stimulation (main effect Time F1,16=1.396, p=.078).

**Discussion:**

Glx was increased during real compared to sham tDCS, and before compared to after stimulation. This could indicate that tDCS overall changes neuronal firing thresholds. However, we did not observe the expected three-way interaction of reduced glutamate levels in the dorsolateral prefrontal cortex and increased glutamate levels in the temporo-parietal cortex. This could be due to the relatively small sample. However, the present data analysis is preliminary and we aim to report findings for a larger dataset.

